# Topoisomerase I but not thymidylate synthase is associated with improved outcome in patients with resected colorectal cancer treated with irinotecan containing adjuvant chemotherapy

**DOI:** 10.1186/1471-2407-9-339

**Published:** 2009-09-24

**Authors:** Ioannis Kostopoulos, Vasilios Karavasilis, Maria Karina, Mattheos Bobos, Nikolaos Xiros, George Pentheroudakis, Georgia Kafiri, Pavlos Papakostas, Eleni Vrettou, George Fountzilas

**Affiliations:** 1 Department of Pathology, Aristotle University of Thessaloniki School of Medicine, Thessaloniki, Greece; 2 Department of Medical Oncology "Papageorgiou" Hospital, Aristotle University of Thessaloniki School of Medicine, Thessaloniki, Greece; 3 Second Department of Internal Medicine-Propaedeutic, Oncology Section, University General Hospital "Attikon", Athens, Greece; 4 Department of Medical Oncology, Ioannina University Hospital, Ioannina, Greece; 5 Department of Pathology, "Ippokration" Hospital, Athens, Greece; 6 Oncology Department, "Ippokration" Hospital, Athens, Greece

## Abstract

**Background:**

Thymidylate synthase (TS) and Topoisomerase I (Topo I) are significant biomarkers in colorectal cancer (CRC). We aimed to study the expression of TS and Topo I in patients with resected CRC who received adjuvant chemotherapy and correlated it with clinical outcome.

**Methods:**

All patients diagnosed with CRC between 1989 and 2007 and treated with adjuvant chemotherapy within Hellenic Cooperative Oncology Group's (HeCOG) protocols, were identified. Archival paraffin-embedded tumor tissues were used for immunohistochemical detection of TS and Topo I. Immunohistochemistry was performed on tissue microarray slides using monoclonal antibodies against TS and Topo I. The results were correlated with survival (OS) and disease free survival (DFS).

**Results:**

A cohort of 498 patients with a median age of 61 years and Dukes' stage B (49%) and C (51%) fulfilled the criteria of the study. All patients received adjuvant 5-FU-based chemotherapy, 38% irinotecan-containing. Positive TS and Topo I expression was found in 43% and 48% of cases, respectively. Five-year OS was 74% and DFS was 68%. In univariate analysis no association of TS and Topo I expression with OS and DFS was identified. In multivariate analysis however, Topo I expression was associated with a reduced risk of death (HR = 0.61, 95% CI 0.42-0.88, p = 0.009). In the irinotecan-treated subgroup, those patients who expressed Topo I had a better OS (HR = 0.47, 95% CI 0.23-0.94, p = 0.033).

**Conclusion:**

Patients with resected CRC expressing Topo I seem to benefit from irinotecan-containing adjuvant chemotherapy. However randomised prospective trials are needed to confirm these results.

## Background

Colorectal cancer is the second most common cause of death from cancer in the western world[1]. Postoperative adjuvant chemotherapy has clearly been shown to improve survival in stage III colon cancer and is now widely accepted as standard therapy[2]. However, the need for adjuvant chemotherapy in stage II disease hasn't been determined as yet, with the exception of patients who are considered to be at high risk for recurrence[2]. Therefore, biological markers that could reliably predict survival are needed.

The antimetabolite, 5-Fluorouracil (5-FU), remains the mainstay of chemotherapy for colorectal cancer (CRC). Adjuvant treatment with 5-FU has been shown to improve survival in stage III colon carcinoma patients by 10%-15%[3]. Survival has further been improved with the addition of the newer drugs oxaliplatin and irinotecan[4]. Although increasing evidence indicates that stage II CRC patients could also have a benefit from 5-FU-based therapies, only a relatively small proportion of patients appear to benefit from this treatment[5]. Therefore, considerable effort has been directed towards the identification of biomarkers that can accurately predict tumor response. These have included intratumoral levels of thymidylate synthase (TS), *TP53 *mutations, microsatellite instability, and chromosomal deletions [6-8].

Thymidylate synthase is essential for de novo DNA synthesis, by catalyzing the conversion of deoxy-uridine monophosphate (dUMP) to deoxy-thymidine monophosphate (dTMP). The TS protein is the target for the antitumor effect of fluoropyrimidines. Inhibition of TS by the 5-FU metabolite fluorodeoxyuridine monophosphate has been identified as the main mechanism of 5-FU action[9]. A number of studies have investigated the relationship between TS protein expression and survival in CRC patients. Although most have reported poor overall survival (OS) and progression-free survival (PFS) with tumors expressing high TS levels, the prognostic value of TS expression between studies has not been established[10].

Topoisomerase I (Topo I) is an essential enzyme with pivotal role in regulating DNA topology[11]. Topo I targeting antineoplastic drugs, such as camptothecins or etoposides, form stable Topo I-DNA cleavage complexes and inhibit Topo I activity by impeding DNA religation[12]. Topo I is expressed in primary colorectal carcinomas and metastases, but its predictive role in patients undergoing anti-Topo I treatment has not been defined as yet[13,14].

In our study we looked in retrospect, whether the immunohistochemical expression of TS and Topo I (which is the target of irinotecan) had any predictive significance in a large cohort of patients with colorectal cancer, who received several schedules of 5-FU-based adjuvant chemotherapy.

## Methods

From the Hellenic Cooperative Oncology Group's (HeCOG) electronic database, all patients with Dukes' stage C and high risk B CRC who received post-operative adjuvant chemotherapy within several treatment protocols between 1989 and 2007 were identified. All patients had participated in the five 5-FU-based adjuvant therapy trials conducted by HeCOG, the results of which have already been published [15-19]. In the first trial, from August 1989 to July 1997, eligible patients with CRC were randomized to receive adjuvant 5-FU/Leucovorin (LV) with or without Interferon alpha (IFNa). In the second trial, from October 1989 to February 1997, patients with rectal cancer were randomized to receive postoperative concomitant radiotherapy and bolus 5-FU chemotherapy with or without additional chemotherapy with 5-FU and high dose LV. In the third randomized trial, in which enrollment took place between October 1999 and December 2007, patients with rectal cancer received radiotherapy and adjuvant 5-FU/LV chemotherapy with or without the addition of irinotecan (CPT-11). Our search also identified a fourth small feasibility study conducted prior to the last one, with irinotecan and 5-FU/LV adjuvant chemotherapy given to patients with rectal cancer. In addition, in a fifth randomized trial published in an abstract form, in which enrollment took place between January 1999 and September 2004, patients with colon cancer received adjuvant 5-FU/Folinic acid chemotherapy with or without the addition of irinotecan. These patients were also included in the total study population. Only patients that received adjuvant chemotherapy were retrieved and selected for the present study. Despite the differences in treatment modalities, the individual trials described above were very similar with regard to the eligibility criteria and evaluation standards employed.

Paraffin-embedded tumor tissue was available from a non-random subset of study participants. Only patients with tumor tissue available in the HeCOG tumor tissue bank were included in the present study. Signed informed consent was obtained for the treatment. Clinical protocols were approved by the institutional review boards of each participating centre. A waiver of consent for the use of archival tissue was provided by the Bioethics Committee of the Aristotle University of Thessaloniki. All patients were censored 5 years after randomization for disease-free survival. Patients were followed for a minimum of 3 years after study randomization.

### Tissue samples

Archival formalin-fixed paraffin-embedded tissue samples were collected from nine different hospitals between 1989 and 2007. Fixation, tissue processing, and storage protocols of these tissue samples were identified and appeared to be highly variable. The H&E sections were reviewed by a pathologist (MB) for the evaluation of tissue adequacy, confirmation of the diagnosis and calculation of the tumor percentage in each case.

### Tissue Microarray (TMA) construction

Twenty-one TMA blocks, containing from 91 to 343 tissue cores, were constructed (by MB) with a manual microarrayer (Beecher Instruments, Sun Prairie, WI, USA) to include 7 cores (0.6 mm diameter) from each CRC case, randomly obtained from both the central part and the invasive front of the tumor. In each TMA block, 7 cores in total, from thyroid, skin and tonsil tissue were included as controls of immunostaining, block orientation and alignment.

### Immunohistochemistry (IHC)

The mouse monoclonal anti-human Thymidylate Synthase antibody (clone TS 106, Neomarkers, Fremont, CA, USA) and the mouse monoclonal anti-human Topoisomerase I antibody (clone 1D6, Newcastle, UK) were used for the IHC assays. Antigen unmasking was performed by heating the slides on a hot plate for 20 minutes with a sodium citrate solution, pH 6.0. After three 5 min washings with TBS the slides were incubated for 1 hour for TS and 10 minutes for Topo I with protein block (Power Block™, BioGenex, San Ramon, CA, USA). The slides were then incubated overnight at 4°C with TS mAb (dilution 1:300), and for 1 hour at ambient temperature with Topoisomerase I mAb (dilution 1:100). After washing of the primary antibody, the slides were incubated in a biotin-free detection system (HRP super sensitive non-biotin detection system, BioGenex) for a total of 40 minutes. The antigen/antibody complex was visualized using diaminobenzidine (BioGenex) as a chromogen. Slides were counterstained with Mayer's hematoxylin. The thyroid and skin tissue cores served as negative IHC controls for the TS and Topo I, while the tonsil tissue as a positive control for Topo I. A known positive colorectal carcinoma case and an ovarian carcinoma case were used as external positive IHC controls for TS and Topo I, respectively.

### Evaluation system

The evaluation of all IHC sections was done simultaneously by two observers, (I.K. and M.B.) according to previously proposed/established criteria with slight modifications [13,20]. Each slide was assigned a score for intensity and staining pattern using a 4-tier system. Intensity score ranged from 0 to 3 (0 = no staining, 1 = weakly positive, 2 = moderately positive, and 3 = strongly positive staining). The staining pattern score, based on the percentage of positive tumor cells, ranged from 0-3 (0 = 0 to 5%, 1 = 6 to 25%, 2 = 26% to 50%, 3 = 51% to 100%). The localization of staining for each protein, cytoplasmic and/or nuclear for TS, and nuclear or nuclear/cytoplasmic or solely cytoplasmic for Topo I was also indicated.

### Statistical analysis

In order to define TS and Topo I expression status, the total score was calculated as the sum of the intensity score and the staining pattern score in each case. Cases with a total score of at least 4, were considered as TS or Topo I positive (high expression tumors), whereas cases with a total score of 0--3 were grouped together and considered as negative or low expression tumors.

Comparisons between subgroups were performed using the χ^2 ^test for categorical characteristics and the Mann-Whitney test for continuous variables. Overall Survival (OS) was calculated as the time from adjuvant chemotherapy initiation to the date of death or the date of last contact. Disease Free Survival (DFS) was calculated as the time from adjuvant chemotherapy initiation to the date of verified disease progression, the date of death or the date of last contact. Deaths without prior verification of disease progression were considered as events in the estimation of DFS. OS and DFS distributions were estimated by the Kaplan-Meier method and comparisons between subgroups were performed using the log-rank test. In order to estimate the hazard ratios of TS or Topo I status for OS and DFS, univariate Cox regression analyses were performed both unadjusted and adjusted for irinotecan chemotherapy.

In the assessment of the predictive value of TS and Topo I, the interaction of each of the biomarkers under study with the irinotecan adjuvant treatment was taken into account. Multivariate Cox models were also fitted, including patient's age, sex (female/male), stage (Dukes' B/C), differentiation grade (well or moderately differentiated/poorly differentiated or undifferentiated), Irinotecan containing adjuvant chemotherapy (No/Yes), Topo I expression (negative/positive), and TS expression (negative/positive). Variables selection was performed using the backwards selection procedure based on the likelihood ratio test. For all comparisons the level of significance was set at a = 0.05.

## Results

### Characteristics of the patients and TS and Topo I status

From a total of 1730 CRC patients registered in our database, 498 cases (229 females and 269 males) were eligible for analysis (Table 1). Median age at diagnosis was 61 years (range 21-79 years). Most patients had rectal (32%) and sigmoid (27%) primaries, predominantly moderately differentiated (72%). Dukes' stage B and C were equally represented. All patients were treated within the previously mentioned therapeutic protocols of HeCOG. In total, 5-FU/LV chemotherapy was given to 286 patients (248 without IFNa and 38 patients with IFNa), whereas 190 patients were treated with irinotecan/5-FU/LV. A small number of patients (3%) were treated with oxaliplatin/5-FU/LV. Approximately 30% of the patients were treated with radiotherapy in the adjuvant setting. The vast majority of these patients (87%) had rectal cancer (Table 1).

**Table 1:** Patient demographics.

	All patients N = 498
**Age **	
Median (range)	63 (21-79)
	**N (%)**
**Sex**	
Female	229 (46)
Male	269 (54)
**Primary site**	
Rectum	161 (32)
Sigmoid	137 (27)
Cecum	69 (14)
Ascending	57 (11)
Descending	37 (7)
Transverse	35 (7)
Unknown	2 (0.4)
**Stage at diagnosis**	
Dukes' B	246 (49)
Dukes' C	252 (51)
**Differentiation Grade**	
Good	67 (14)
Moderate	357 (72)
Poor	62 (12)
Undifferentiated	3 (1)
Unknown	9 (2)
**Adjuvant radiotherapy**	
No	325 (65)
Yes	143 (29)
Unknown	30 (6)
**Adjuvant chemotherapy**	
5FU/LV/Irinotecan	190 (38)
5FU/LV ± IFNα	286 (57)
5FU/LV/Oxaliplatin	17 (3)
Other	5 (1)

The patients included in the analysis had a significantly higher percentage of Dukes' stage B_2 _at diagnosis (49% vs. 43%, χ^2 ^test, p = 0.034) than the protocol patients excluded due to inability to obtain tissue for IHC evaluation. The rest of the patient characteristics presented in Table 1 (age, sex, primary site, and differentiation grade) were not significantly different.

**Figure 1:**
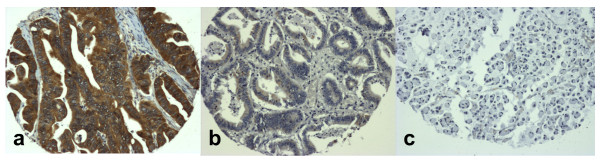
**Immunohistochemical detection of TS in TMA cores of colorectal carcinoma cases**. Strong cytoplasmic staining, graded as positive with a total score of 6/6 (a); mild focal cytoplasmic staining, graded as negative with a total score of 2/6 (b); absence of staining in a signet-ring cell carcinoma, with a total score of 0/6 (c). (Magnification: ×100).

**Figure 2:**
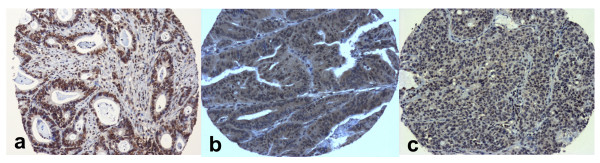
**Immunohistochemical detection of Topo I in TMA cores of colorectal carcinoma cases**. Strong nuclear staining, graded as positive with a total score of 6/6 (a); moderate, diffused, predominantly cytoplasmic staining, graded as positive with a total score of 5/6 (b); mild focal nuclear and cytoplasmic staining, graded as low expression/negative with a total score of 2/6 (c). (Magnification: ×100).

High TS expression (Figure 1) was found in 212 (43%) of the cases, whereas in 2% of the patients, expression status could not be assessed due to inadequate material (Table 2). High expression of Topo I (Figure 2) was found in 238 (48%) of cases. In 3% of the patients the staining for Topo I was inappropriate for assessing protein status (Table 2).

**Table 2:** Immunohistochemical expression of thymidylate synthase (TS) and topoisomerase I (Topo I) in tumor tissue samples (N = 498).

TS score	N	%	
0	157	32	
1	1	0.2	Negative/Low
2	47	9	expression
3	73	15	

4	105	21	
5	60	12	Positive/High
6	47	9	expression
Non Evaluable	8	2	

Topo I score			

0	170	34	
2	19	4	Negative/Low
3	54	11	expression

4	126	25	
5	80	16	Positive/High
6	32	6	expression
Non Evaluable	17	3	

### Relation between TS and Topo I status and survival

**Figure 3:**
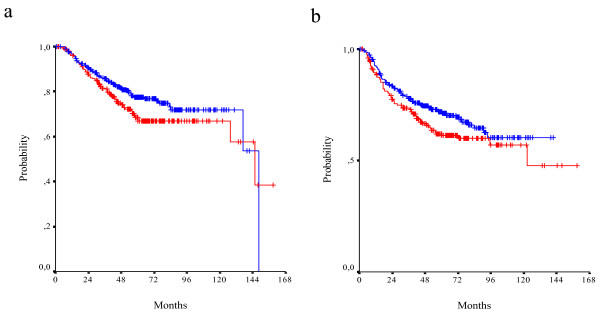
**OS (a) and DFS (b) in TS positive (red line) and TS negative (blue line) patients (log-rank p = 0.100 and p = 0.079, respectively)**.

**Figure 4:**
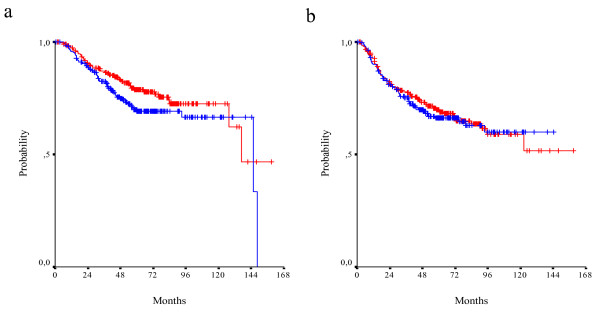
**OS (a) and DFS (b) in Topo I positive (red line) and Topo I negative (blue line) patients (log-rank p = 0.079 and p = 0.691, respectively)**.

With a median follow-up of 63.5 months, 160 relapses and 126 deaths were recorded. The 3-year OS rate was 84%, the 5-year OS rate 74% and the median survival was 145.7 months. The 3-year DFS rate was 76% and 5-year DFS rate 68%. In univariate analysis, a non-significant trend for a poor survival in TS positive patients was noticed (5-year OS rate 68% vs 77%, median survival 146 months vs 148 months, log-rank p = 0.100). A similar trend was noticed for DFS (5-year DFS rate 62% vs 72%, median DFS 123 months vs not reached yet, log-rank p = 0.079) (Figure 3). With regards to Topo I expression, there was a trend for better survival in patients who expressed Topo I (5-year OS rate 79% vs 70%, median survival 146 months vs 137 months, p = 0.079). For DFS no such trend was detected (5-year DFS rate 70% vs 66%, median survival not reached yet, log-rank p = 0.691) (Figure 4).

In multivariate analysis (Table 3), positive Topo I expression was found to be significantly associated with improved overall survival (HR = 0.61, 95% CI 0.42-0.88, wald p = 0.009), but no such association was seen with DFS (HR = 0.80, 95% CI 0.58-1.11, wald p = 0.18). TS expression was not found to be an independent prognostic factor for OS or DFS.

**Table 3:** Multivariate Cox regression analysis.

	OS			DFS		
	HR	95% CI	p	HR	95% CI	p
**Stage**						
Dukes B	1	-		1	-	
Dukes C	2.75	1.84-4.12	<0.001	2.46	1.74-3.48	<0.001
**Differentiation grade**						
Well or Moderate	1	-		1	-	
Poor	2.51	1.60-3.94	<0.001	2.56	1.73-3.79	<0.001
**TS expression**						
Negative/Low expression	1	-		1	-	
Positive/High	1.37	0.94-1.97	0.098	1.32	0.95-1.82	0.097
**Topo I expression**						
Negative/Low expression	1	-		1	-	
Positive/High	0.61	0.42-0.88	0.009	0.80	0.58-1.11	0.180
**CPT-11 treatment**						
No	1	-		1	-	
Yes	0.78	0.52-1.16	0.216	0.79	0.56-1.12	0.187

Interaction between Topo I and TS adjusted for all factors included in the multivariate analysis was not significant either for OS or DFS (wald, p = 0.632 and p = 0.798 respectively). However, if looking at each of the four groups seperately, (TS/Topo I both positive vs TS negative/Topo I positive vs TS positive/Topo I negative vs TS/Topo I both negative), the TS positive/Topo I negative group was associated with shorter OS compared to the other three groups (wald p = 0.036). Adjusting for all other factors (stage, grade and irinotecan treatment) this result remains significant (wald p = 0.022).

### Relation between TS and Topo I status and benefit from adjuvant irinotecan containing chemotherapy

**Figure 5:**
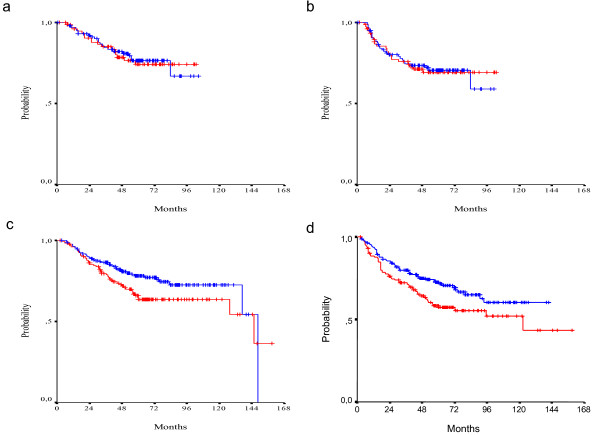
**OS (a) and DFS (b) in TS positive (red line) and TS negative (blue line) CPT-11 treated patients (log-rank p = 0.868 and p = 0.937, respectively)**. OS (c) and DFS (d) in TS positive (red line) and TS negative (blue line) non-CPT-11 treated patients (log-rank p = 0.062 and p = 0.035, respectively).

In total, 190 patients were treated with irinotecan and 5-FU/LV chemotherapy. There were no differences in survival between patients who received irinotecan and those who did not. However median OS and DFS have not been reached yet. Interaction of irinotecan containing adjuvant chemotherapy with the two biomarkers assessed was not shown to be significant. Nevertheless, in the absence of irinotecan treatment, positive TS expression tends to have a negative effect on survival (HR = 1.50, 95% CI 0.98-2.30, wald p = 0.068). This effect is not seen in CPT-11 treated patients (HR = 1.06, 95% CI 0.56-2.00, wald p = 0.857). Results for DFS were similar. In the subgroup of irinotecan treated patients the effect of TS expression was not statistically significant (HR = 1.02, 95% CI 0.59-1.77, wald p = 0.946), while in the absence of irinotecan, TS was associated with a worse DFS (HR = 1.50, 95% CI 1.03-2.19, wald p = 0.036) (Figure 5).

**Figure 6:**
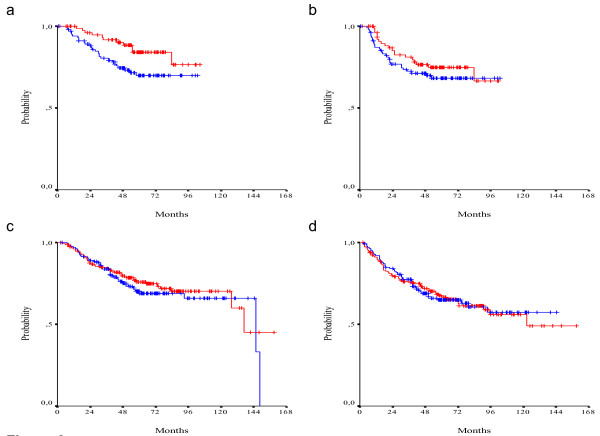
**OS (a) and DFS (b) in Topo I positive (red line) and Topo I negative (blue line) CPT-11 treated patients (log-rank p = 0.028 and p = 0.323, respectively)**. OS (c) and DFS (d) in Topo I positive (red line) and Topo I negative (blue line) non-CPT-11 treated patients (log-rank p = 0.478 and p = 0.945, respectively).

On the other hand, positive Topo I expression seems to have a prophylactic effect in terms of OS in irinotecan treated patients by reducing the hazard for death by 53% (HR = 0.47, 95% CI 0.23-0.94, wald p = 0.033). In the absence of irinotecan, Topo I expression has no effect on OS (HR = 0.86, 95% CI 0.55-1.32, wald p = 0.481). Effect of Topo I expression on DFS was not shown to be statistically significant in either irinotecan treated or non-treated patients (HR = 0.76, 95% CI 0.43-1.34, wald p = 0.338 and HR = 1.02, 95% CI 0.69-1.50, wald p = 0.925, respectively) (Figure 6).

## Discussion

In our study we failed to demonstrate an association between TS and clinical outcome in patients with resected CRC treated with adjuvant chemotherapy. However, a non-significant trend for poor OS and DFS was seen in TS positive patients. High TS expression levels have generally been associated with poor overall survival in CRC. Most of the studies to date show that high levels of TS correlate with lower response rates in patients with advanced disease treated with fluoropyrimidine-based therapies [21-23].

In the adjuvant setting, a meta-analysis, which included 2610 patients with resected CRC, found a hazard ratio of recurrence of 1.35 for high TS, although the adjuvant treatment status of patients in many of the studies was not well defined[24]. Only few trials had a sufficiently large sample to explore the efficacy of fluorouracil-based adjuvant chemotherapy according to TS expression. Allegra et al. failed to demonstrate a consistent and significant association between TS and either OS or DFS in 465 patients with Dukes B and C disease[20]. Moreover, Popat et al., in a large prospective trial of the prognostic value of the molecular markers TS and p53 in resected CRC, similarly failed to demonstrate such an association of TS expression and clinical outcome in the adjuvant setting[25].

In controversy, Kornmann et al. showed, in a cohort of 309 patients with resected CRC treated with adjuvant 5-FU therapy, that those with high TS survived longer than those with low TS[26]. Recently, Jansen et al. found that high TS gene copy numbers were associated with a significantly higher risk of recurrence and death in patients with colorectal cancer treated with adjuvant 5-FU chemotherapy[27]. It appears therefore, that the prognostic role of TS expression in CRC remains controversial in the adjuvant setting. Several hypotheses have been suggested to explain this occurrence, including differences in TS levels in primary tumors and locoregional lymph node metastases, modulation of 5-FU resistance by other molecules such as p53 and DPD, differences in 5-FU scheduling, as well as methodological problems and biases in the assessment of TS in individual studies[10,25]. In addition, no relationship between TS protein expression and TS enzyme activity, measured by FdUMP-binding or TS catalytic assays, was found in CRC patients[28], which may be another factor contributing to the conflicting data.

Our finding of a significant association of high TS expression with shorter DFS in a subgroup of patients not treated with irinotecan adds to the controversy. One possible explanation is that the predictive value of TS can only be detected in the non-irinotecan treated group, since there is no data available connecting TS expression with responsiveness to irinotecan. However the possibility that this is a result of the increased heterogeneity, with regards to the administered chemotherapy regimens in this group, cannot be excluded.

While there are data regarding the prognostic role of TS in the adjuvant setting in patients with CRC, little is known about the possible role of Topo I. Topo I is the target of the active irinotecan metabolite SN 38 and may therefore be a plausible predictive marker for irinotecan containing chemotherapy[14]. Expression of Topo I varies between 43% and 51% in published series, while Topo I enzymatic activity has been found highly variable between malignant and normal tissue of patients with CRC[13,29,30]. In our study, in a multivariate analysis, it turned out that Topo I expression was an independent prognostic factor for survival by reducing the hazard ratio of death by 39%. In a subgroup analysis, it seemed that this survival benefit applies only to patients who expressed Topo I and were treated with adjuvant irinotecan containing chemotherapy.

Nevertheless, we were not able to show statistical benefit in DFS from the addition of irinotecan. We postulate that possible explanations might be the relatively small number of patients that received adjuvant irinotecan chemotherapy, the retrospective nature of our study and the heterogeneity of the patient population. Several phase III randomized trials did not show any incremental benefit from the addition of irinotecan to 5-FU/LV adjuvant chemotherapy in patients with colorectal cancer[18,31]. Whether Topo I expression is a favorable prognostic marker by itself and whether this can further be improved by adding irinotecan in the adjuvant treatment of this population still remains unanswered.

Very recently, Braun and colleagues investigated a panel of possible biomarkers for chemotherapy efficacy in a large cohort of patients with metastatic colorectal cancer, receiving chemotherapy within the FOCUS trial[32]. Between several molecules tested, they found that high levels of Topo I (assessed by similar to our study IHC methods) were associated with a major survival benefit from first line chemotherapy regimens containing either irinotecan or oxaliplatin, corresponding to a median survival advantage of 5.3 months. Nevertheless, there is still lack of information regarding the prognostic role of Topo I in the adjuvant setting.

## Conclusion

Our results failed to demonstrate an association between TS and clinical outcome in patients with CRC treated with various regimens of 5-FU based adjuvant chemotherapy. On the other hand we have shown that Topo I is an independent prognostic factor of survival. Adjuvant irinotecan containing chemotherapy might confer a survival advantage in CRC patients expressing Topo I, but this is still an open issue amenable to further investigation.

## Competing interests

The authors declare that they have no competing interests.

## Authors' contributions

IK conceived of the study, participated in its design, carried out the immunoassays, evaluated and interpreted the data and drafted the manuscript. VK participated in its design and coordination and drafted the manuscript, equally contributed with IK. MK participated in its design and coordination and helped to draft the manuscript. MB participated in its design and coordination, carried out the immunoassays, contributed to evaluation and interpretation of the data and helped to draft the manuscript. NX participated in its design and coordination and helped to draft the manuscript. GP participated in its design and coordination and helped to draft the manuscript. GK participated in its design and coordination and helped to draft the manuscript. PP participated in its design and coordination and helped to draft the manuscript. EV participated in its design and coordination and helped to draft the manuscript. GF conceived of the study, participated in its design, evaluated and interpreted the data and helped to draft the manuscript. All authors read and approved the final manuscript.

## Pre-publication history

The pre-publication history for this paper can be accessed here:

http://www.biomedcentral.com/1471-2407/9/339/prepub
